# The characteristics and outcomes of vascular abnormalities involving limbs in children: a retrospective analysis of 35 cases

**DOI:** 10.3389/fped.2026.1841905

**Published:** 2026-07-14

**Authors:** Haiyin Zhou, Xiangyu Yang, Jialong Nie, Zixiao Wang, Ting Lei, Kun Liu, Guanghui Zhu

**Affiliations:** 1 General Emergency Ward, The Affiliated Children’s Hospital of Xiangya School of Medicine, Central South University (Hunan Children’s Hospital), Changsha, Hunan, China; 2 Department of Orthopedics, The Affiliated Children’s Hospital of Xiangya School of Medicine, Central South University (Hunan Children’s Hospital), Hunan Provincial Key Laboratory of Pediatric Orthopedics, Changsha, Hunan, China; 3 The School of Pediatrics, University of South China, Changsha, Hunan, China; 4 Major Disease Research Center, Furong Laboratory, Changsha, Hunan, China

**Keywords:** children, diagnosis, limbs, surgical treatment, vascular abnormality

## Abstract

**Objective:**

To explore the clinical characteristics, diagnostic criteria, and surgical outcomes of vascular abnormalities in the limbs of children.

**Methods:**

The clinical data of children diagnosed with pathologically confirmed limb vascular abnormalities at Hunan Children's Hospital from January 2007 to December 2025 were retrospectively analyzed. Demographic data including pathological types, lesion locations, clinical manifestations, surgical procedures and therapeutic outcomes were summarized.

**Result:**

Among the 35 children, 18 (51.4%) were male and 17 (48.6%) were female. The age at surgery was 1.8 to 14.2 years, with an average of (7.4 ± 3.2) years. Lesions involved the upper limbs in 16 cases and the lower limbs in 19 cases. The main clinical manifestations were localized masses in 32 cases (91.4%), pain in 19 cases (54.3%), joint dysfunction in 3 cases (8.6%), limping in 2 cases (5.7%), and skin ulcers in 1 case (2.9%). The size of the masses ranged from 1.0 to 9.6 cm, with an average of (3.8 ± 2.3) cm. Three cases (8.6%) had a history of multiple interventional embolization or sclerotherapy injections. Two cases (5.7%) had undergone previous resection surgery in other hospitals. All cases were treated with surgical excision in our department. Pathological types revealed venous malformation (VM) in 14 cases (40.0%), arteriovenous malformation (AVM) in 12 cases (34.3%), lymphangioma (LM) in 5 cases (14.3%), and intermuscular hemangioma (IH) in 4 cases (11.4%). All patients were followed up, with an average postoperative follow-up period of 0.3 to 18.2 years (mean: 6.4 ± 4.5 years). One case developed finger extension restriction and scar contracture, two cases had elbow extension restriction, two cases experienced local recurrence, and there were no cases of skin necrosis or infection. The surgical complication rate was 11.4%, and the recurrence rate was 5.7%. There was no significant difference in complication rate and recurrence rate among different pathological types (*P* = 0.283 and *P* = 0.176 respectively).

**Conclusion:**

The main clinical manifestations of vascular abnormalities in pediatric limbs include local masses and pain. Surgical resection can be adopted for localized, well-demarcated limb vascular malformations as well as cases failed to interventional therapy, yielding promising clinical outcomes with a low recurrence rate and reliable preservation of limb function.

## 1 Introduction

Vascular malformations occur with an incidence of 1.5% in the population (1, 2). Limb involvement of vascular malformations mainly include venous malformation (VM), fibroadipose vascular anomaly (FAVA), arteriovenous malformation (AVM), and angiomatosis of soft tissue (AST) (3–5). As children grow, lesions may gradually enlarge, compressing the surrounding muscles, nerves, and bones, resulting in local masses, persistent pain, limb dysfunction, and even disuse atrophy, which adversely affect physical and mental health. Due to deep lesion locations and nonspecific early symptoms of certain vascular malformations, such conditions could be misdiagnosed as growing pain, muscle strain, lipoma and other disorders clinically, with the misdiagnosis rate ranging from 18.8% to 35.2% (6, 7).

Currently, a stratified diagnostic system of vascular abnormalities involving limbs centered on imaging examinations has been established in clinical practice, where ultrasound, MRI, DSA, and genetic testing can effectively achieve disease classification and condition assessment. The treatment paradigm has also shifted from traditional open surgery to an individualized comprehensive approach combining minimally invasive intervention, surgical reconstruction, targeted drugs, and rehabilitation intervention (1, 6). However, at this stage, clinical practice still faces challenges such as a high misdiagnosis rate for rare subtypes and a high recurrence rate in complex cases. Standardized treatment for vascular malformations in children's limbs has not been unified, and there is a lack of large case series demonstrating diagnostic and treatment principles. This study retrospectively analyzed the clinical data of 35 children in a single center, systematically summarizing the clinical characteristics and therapeutic advances of vascular abnormalities in the limbs of children, and aimed to provide evidence for standardized clinical diagnosis and treatment.

## 2 Materials and methods

### 2.1 Study subjects and ethical approval

This study has been approved by the Ethics Committee of Hunan Children's Hospital. Children with limb vascular abnormalities admitted to the Orthopedics Department of Hunan Children's Hospital from January 2007 to December 2025 were selected as the research subjects. All included study cases completed informed consent. Retrospective analysis of demographic data from inpatient and outpatient data was performed, recording the pathological types, locations, clinical symptoms, disease duration, surgical methods, and treatment outcomes of vascular abnormalities.

### 2.2 The inclusion criteria

① Age ≤ 18 years old; ② The vascular abnormalities were located in the limbs and were confirmed by surgery and pathological examination; ③ The postoperative pathology meets the diagnostic criteria of the “ISVI-IUA consensus document diagnostic guidelines of vascular anomalies: vascular malformations and hemangiomas” (8); ④ Detailed clinical data were available, including medical history, physical examination, imaging examination, surgical records, pathological reports, and follow-up data; ⑤ Follow up period ≥ 3 months.

### 2.3 The exclusion criteria

① Combined vascular malformations in the head and neck, chest, abdomen, and other areas; ② Complicated with malignant tumors, genetic diseases (such as PHACE syndrome, CLOVES syndrome), or dysfunction of important organs such as the heart, liver, and kidneys; ③ Incomplete clinical data to complete efficacy evaluation; ④ Complicated with infectious diseases or abnormal coagulation function.

### 2.4 Statistical methods

SPSS 26.0 (International Business Machines Corporation, USA) was used for statistical analysis. For measurement data, mean and standard deviation were used to represent it. Comparisons between groups of categorical data were performed using Fisher's exact test. An alpha level of 0.05 was used to define statistical significance.

## 3 Results

### 3.1 Cohort characteristics

Among the 35 patients, 18 (51.4%) were male and 17 (48.6%) were female; lesions affected the left limb in 19 patients and the right limb in 16 patients. Seven lesions were located in the hands, and four were located in the feet. The patients’ age at initial consultation ranged from 1.8 to 14.2 years, with an average of (7.4 ± 3.2) years. In this study, the interval between initial consultation and surgery was restricted to 2–4 days; accordingly the date of the initial consultation for all 35 patients was nearly coincided with the date of surgery. There were 16 cases of lesions involving the upper limbs and 19 cases involving the lower limbs, among which 11 cases involving the hands or feet. For 16 cases with upper limb lesions (2 presented multifocal lesions spanning two anatomical sites), the number of cases in each sub-location were upper arm (1 case), elbow (3 cases), forearm (4 cases), and hand/wrist (10 cases). For lower limb lesions (19 cases), the sub-location details were thigh (3 cases), knee (8 cases), calf (3 cases) and ankle/foot (5 cases).

### 3.2 Clinical symptoms

Local masses were the initial symptoms in 32 cases (91.4%), with lesions demonstrating soft or tough texture, ill-defined boundaries, and poor mobility. Nineteen cases (54.3%) reported local pain, predominantly persistent dull or distending pain that worsened with physical exertion. Three cases (8.6%) had joint dysfunction, manifested as limited flexion and extension of fingers, elbows, and wrists. Two patients presented with symptoms of limping (5.7%). One case (2.9%) had skin ulcer over the elbow.

### 3.3 Previous treatment

Three cases (8.6%) had a history of multiple interventional embolization or sclerotherapy injections. Among them, one case had undergone four rounds of left upper limb lymphangioma perfusion with lauromacrogol, but experienced unsatisfactory therapeutic outcomes. One case underwent percutaneous super selective arterial angiography and transcatheter embolization of left upper limb arteriovenous malformation. One case of distal right forearm venous malformation underwent one image-guided percutaneous sclerotherapy and one transcatheter embolization. None of these three cases exhibited significant lesion regression or symptomatic improvement following interventional therapy and sclerosing agent injection. Two cases (5.7%) had undergone surgical resection in other hospitals previously, yet experienced postoperative recurrence and were referred to our department for salvage management. Patient demographics characteristics were shown in Table 1.

**Table 1:** Patient details.

Case no.	Age (yrs)	Follow up (yrs)	Gender	Side	Location	Symptoms	Previous surgery	Lesion size (cm)	Tissue involvement	Pathology	Function	Recurrence	Other complications
1	4.9	1.2	Male	Right	foot	Mass, Pain	N	3.5	muscle	VM	normal	N	N
2	6.5	6.2	Male	Left	index and middle finger	Mass, Pain	N	1	subcutaneous	VM	normal	N	N
3	11.1	2.5	Male	Right	wrist	Mass, Pain, JD	transcatheter embolization	1.9	muscle	VM	normal	N	N
4	12.3	0.3	Male	Right	knee	Pain	N	3.8	muscle	VM	normal	N	N
5	9.5	5.4	Female	Right	Thumb and palm	Mass, Pain	N	2.5	muscle	VM	normal	Y	N
6	6	8.8	Female	Right	thumb	Mass	N	1.2	subcutaneous	VM	normal	N	N
7	5.3	7.5	Female	Left	Little finger	Mass, Pain	N	1	subcutaneous	VM	normal	N	N
8	5.6	1.4	Male	Right	thumb and palm	Mass	N	1.1	muscle	VM	normal	N	N
9	7.8	1.8	Female	Left	Index finger	Mass	N	1.5	subcutaneous	VM	normal	N	N
10	9.1	2.7	Female	Left	toe	Mass, Pain	Lesion resection	1.5	subcutaneous	VM	normal	Y	N
11	8.1	3	Male	Left	knee	Pain	N	5.5	muscle	VM	normal	N	N
12	4.5	4.4	Female	Right	calf	Mass, pain	N	1.5	muscle	VM	normal	N	N
13	3	5.5	Male	Left	ankle	Mass	N	3	muscle	VM	normal	N	N
14	8.8	6.6	Female	Left	foot	Mass, Pain, limping	N	5.8	muscle	VM	normal	N	N
15	4.3	8	Female	Right	Finger	Mass, JD	N	3	subcutaneous	AVM	extension restriction	N	scar contracture
16	12.3	0.4	Male	Right	knee	Pain	N	3.8	subcutaneous	AVM	normal	N	N
17	9	9.1	Male	Left	thigh	Mass, Pain	N	7.9	subcutaneous	AVM	normal	N	N
18	14.8	0.3	Female	Left	Forearm, elbow	Mass, Pain, JD	transcatheter embolization and Sclerotherapy	5.4	muscle	AVM	extension restriction	N	N
19	7	15	Male	Right	knee	Mass	N	6.7	muscle	AVM	normal	N	N
20	5.8	13.5	Female	Left	calf	Mass	N	5.5	subcutaneous	AVM	normal	N	N
21	10.8	2.8	Female	Right	knee	Mass, Pain	N	3.3	muscle	AVM	normal	N	N
22	8.1	2.2	Male	Right	forearm	Mass	N	3.6	subcutaneous	AVM	normal	N	N
23	6.3	18.2	Male	Left	knee	Mass	N	5	subcutaneous	AVM	normal	N	N
24	5.8	8.1	Male	Left	thigh	Mass, Pain, limping	N	2.5	muscle	AVM	normal	N	N
25	2.5	1.3	Male	Left	elbow	Mass	Lesion resection	1	subcutaneous	AVM	normal	N	N
26	1.8	4.8	Female	Left	knee	Mass	N	3.1	subcutaneous	AVM	normal	N	N
27	9.4	9.1	Female	Right	knee	Mass, Pain	N	5.1	muscle	LM	normal	N	N
28	5	8.5	Male	Left	wrist	Mass, Pain	Sclerotherapy for 4 times	3	subcutaneous	LM	normal	N	N
29	6	7.5	Male	Right	forearm	Mass, Pain	N	9.6	subcutaneous	LM	normal	N	N
30	14.2	7.2	Male	Left	thigh	Mass, Pain	N	4.1	muscle	LM	normal	N	N
31	7	12.7	Female	Right	upper arm, forearm	Mass, skin ulcer	N	2.8	subcutaneous	LM	extension restriction	N	N
32	8	8.5	Male	Left	elbow	Mass	N	9.6	muscle	IH	normal	N	N
33	6.7	8.4	Female	Left	opisthenar	Mass	N	4.3	muscle	IH	normal	N	N
34	2	7.2	Female	Right	calf	Mass	N	3.8	muscle	IH	normal	N	N
35	10.8	13	Female	Left	foot	Mass	N	4.4	muscle	IH	normal	N	N

JD, joint dysfunction; VM, venous malformation; AVM, arteriovenous malformation; LM, lymphangioma; IH, intermuscular hemangioma; Y, yes; N, no.

### 3.4 Auxiliary examination

Multiple imaging studies, Ultrasound (US) combined with Magnetic Resonance Imaging (MRI) or Computed Tomography (CT), were adopted in cases where single imaging modality failed to fully evaluate the lesion. In our cohort, US was done in all 32 cases (91.4%), MRI was performed in 34 cases (97.1%), and CT scan was completed in 5 cases (14.3%).

Ultrasound findings: Among the 35 patients, 28 showed hypoechoic heterogeneous masses with Adler grading of blood flow signal from 0 to I (no or minimal blood flow signal); Four cases of venous malformation demonstrated a wide and hypoechoic area with septa with compressible features, and the blood flow signal was classified as Grade II by Adler; All four cases of venous malformation showed punctate strong echoes (venous stones) with posterior acoustic shadows.

MRI findings: 30 cases of lesions showed equal/low signal on T1WI, high signal on T2WI, and increased signal intensity in the lipid suppression sequence. Four cases of venous malformation showed high signal of venous stones (low signal on T1WI and T2WI with clear boundaries), and three cases were accompanied by bleeding and thrombosis (hyperintense signals on both T1WI and T2WI); 18 cases had clear boundaries with surrounding muscle tissue, and 3 cases exhibited diffuse growth and adhesion with surrounding muscle fibers. The size of the mass is 1.0–9.6 cm, with an average of (3.8 ± 2.3) cm.

CT findings: Five cases completed CT examination and all lesions manifested low-density lesions in soft tissue with CT values of 10–30 HU. Enhanced scans showed mild to moderate enhancement (with an increase of 10–20 HU in CT values). According to the image studies, 16 cases (45.7%) were subcutaneous lesions, and 19 cases (54.3%) were intramuscular lesions (including the 4 cases of intermuscular hemangiomas).

### 3.5 Surgical resection indication and method

The surgical indications for pediatric limb vascular abnormalities included persistent pain, limb functional impairment, obvious mass, and refractory conservative/minimally invasive treatment.

The resection surgery was performed under general anesthesia with a tourniquet. An “S” or “Z” shaped incision was designed based on the surface projection of the lesion, and the lesion was fully exposed by cutting through the skin, subcutaneous tissue, and deep fascia layer by layer. Carefully dissection was performed along the edge of the lesion to avoid entering the lesion and causing massive bleeding. Attention should be paid to protect important blood vessels and nerves such as the median nerve, ulnar nerve, radial nerve, femoral artery, and popliteal artery. Aberrant feeding vessels were transected and ligated. For lesions with unclear boundaries, a small amount of surrounding normal muscle tissue (0.5 cm beyond the lesion margin) can be appropriately removed.

The postoperative pathological types were venous malformation (VM) in 14 cases (40.0%) consisting of 9 intramuscular or 5 subcutaneous lesions, arteriovenous malformation (AVM) in 12 cases (34.3%), lymphangioma (LM) in 5 cases (14.3%), and intermuscular hemangioma (IH) in 4 cases (11.4%).

### 3.6 Outcomes and complications

**Figure 1:**
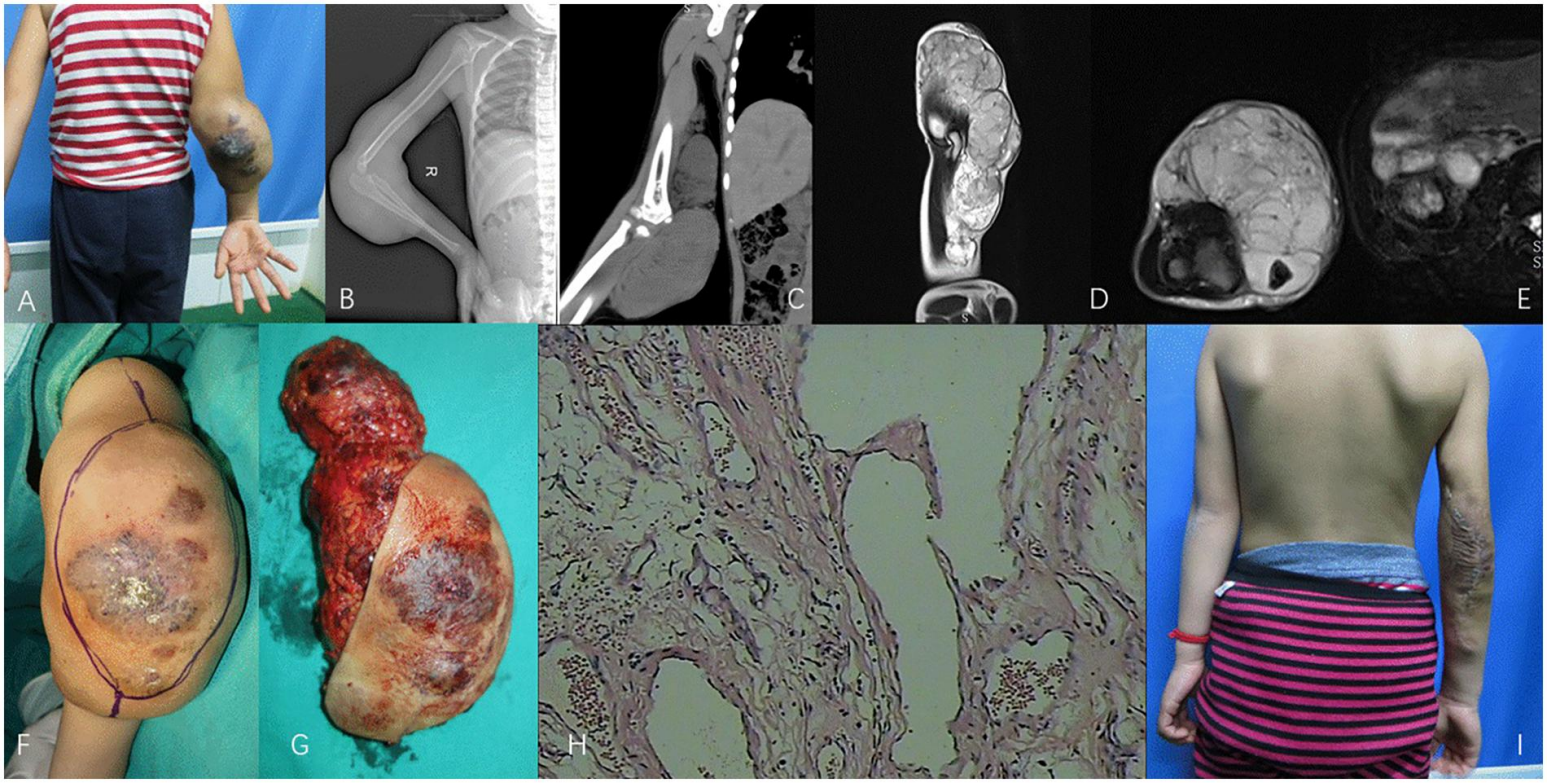
Treatment process for a 7-year-old girl with a huge lymphangioma in the right elbow. **(A)** Preoperative photo shows the appearance of the vascular malformation, as well as skin ulcers and scabs. **(B,C)** Preoperative x-rays and CT scans indicate increased density in the soft tissue of the right elbow. **(D,E)** Preoperative MRI showed a long T1 and T2 signal intensity in the right elbow, suggesting lymphangioma with bleeding. **(F,G)** Intraoperative incision design and gross view of the lesion after complete lymphangioma resection. **(H)** Pathological examination confirms a vascular malformation complicated with infection and bleeding, predominantly consisting of cystic lymphangioma components. **(I)** Postoperative appearance of the right upper limb at the 2-month follow-up.

All lesions were completely resected, and postoperative pain was fully relieved. Postoperative follow-up was conducted for 0.3 to 18.2 years, with an average of (6.4 ± 4.5) years. Most cases (32 cases, 91.4%) had a follow-up period of more than 1 year, and 21 cases (60.0%) had a follow-up period of more than 5 years. The surgical related complications including joint dysfunction in 3 patients and scar contracture in 1 patient, with an incidence of 11.4%. One case of arteriovenous malformation in the middle finger of the right showed scar contracture on the palmar side of the finger and restricted finger extension postoperatively, and subsequently underwent scar release combined with skin grafting for functional improvement. Two cases with extension restriction of elbow joint occurred. However, there was still 20° extension restriction after systemic rehabilitation. Given the negligible impact on daily activities, no further treatment has been given. No significant difference in complication rate was observed among pathological types (*P* = 0.283). Two VM cases (5.7%) had local recurrence (one involved in thumb, one in toe), and both patients underwent secondary surgical resection. No significant inter-group difference in recurrence rates was identified according to pathological classification (*P* = 0.176). There were no case of skin necrosis or infection (Table 1). We present a typical case in Figure 1.

## 4 Discussion

Limb vascular malformations in children are predominantly diagnosed at the preschool and school-aged stages. The average age of the children in this study group was (7.4 ± 3.2) years, which is consistent with previous international findings of (7.8 ± 3.5) years, indicating that this age group is a high-risk period for vascular abnormalities in children (9). This phenomenon may be associated with increased physical activity in children of this age, and the clinical symptoms gradually appear and are discovered after the diseased soft tissue is stretched and stimulated; In addition, some lesions in infants and young children may not be diagnosed early due to small size and subtle early manifestations (10). In terms of gender distribution, the male to female ratio in this study was close to 1.06:1, with no significant gender-based difference. In our cohort, the lesions were more common in the wrist/hand, forearm, and elbow in the upper limbs, and in the calf, ankle and foot in the lower limbs. This anatomical distribution may be attributed to the rich muscle tissue and dense vascular and lymphatic networks in pediatric limbs.

Ultrasound features simplicity, non-invasiveness, and cost-effectiveness, and can serve as a preferred preliminary screening method for vascular abnormalities in children's limbs (1). The typical manifestations are low echo heterogeneous masses and decreased blood flow signals. For superficial lesions, ultrasound can also guide minimally invasive treatments such as injection of sclerosing agents. CT examination is mainly applied to evaluate association between lesions and adjacent bones, as well as the presence of bone involvement, bone destruction or bone hyperplasia, thus providing diagnostic and therapeutic references for children with concomitant bone lesions (11). MRI can clearly display the location, range, size, signal characteristics, and adjacent relationships with surrounding muscles, blood vessels, nerves, and bones (12). Pathological examination combined with immunohistochemical detection enables accurate classification of pathological subtypes of vascular abnormalities in the limbs. In this study, VM constituted the predominant pathological type, followed by AVM.

Minimally invasive intervention therapy, including injection of sclerosing agents and arterial embolization, has become an important component of the treatment of vascular abnormalities in pediatric limbs, especially for low-flow malformations such as venous malformations, lymphatic malformations, as well as lesions with large size or deep location (13, 14). The injection of sclerosants has an effective rate of 54%—83% for VM, among which polyglycol has become the preferred sclerosant for children with intramuscular vascular malformations due to its low irritability and fewer complications; For superficial, small and medium-sized VM, simple sclerosant injection can achieve curative effects; For large or complex lesions, sclerosant injection can be applied as neoadjuvant therapy prior to surgery to reduce lesion volume and decrease surgical difficulty (15). However, it is noteworthy that sclerotherapy for VM carries a risk of secondary muscle contracture, and large-scale, long-term follow-up evaluations of its efficacy remain lacking. Arterial embolization is mainly used for large VM or FAVA with abundant blood supply (16, 17). Through super selective arterial catheterization and injection of embolization materials, the blood supply of the lesion is blocked, causing the lesion to shrink and ischemic necrosis, which facilitates subsequent surgical resection. Targeted drug therapy provides a new approach for the treatment of vascular malformations in pediatric limb, and is particularly applicable to complex subtypes or lesions that cannot be completely resected surgically (18). Sirolimus, as an mTOR inhibitor, can inhibit endothelial cell proliferation and angiogenesis, and has demonstrated satisfactory clinical efficacy and safety in pediatric patients with complex VM for whom conventional standard therapy is insufficient (19).

Treatment for pediatric limb vascular abnormalities are formulated according to lesion size, depth, anatomical location, clinical symptoms and limb function of children. The core therapeutic goals are to eradicate lesions, alleviate symptoms, preserve limb function and improve cosmetic appearance. Surgical resection serves as a major mainstream treatment for vascular abnormalities in children's limbs, especially for focal, well-delineated malformations and that with ineffective conservative/minimally invasive treatment (20–22). Some of the surgical cases in our cohort were ineffective after embolization or sclerotherapy, while others had large masses or lesions involving the fingers and toes, all of which were eligible for surgical resection. The core principle for surgical treatment is to completely remove the lesion and maximally preserving surrounding important blood vessels, nerves, muscles, and normal soft tissues, so as to reduce postoperative limb dysfunction. Preoperative comprehensive imaging evaluation, which clarifies the extent, size, and adjacent relationship of the lesion with surrounding tissues, is a prerequisite for improving surgical resection rate and reducing surgical risks; The adoption of “S-shaped” or “Z-shaped” surgical incisions enables adequate lesion exposure and prevents postoperative scar contracture secondary to linear incisions. Meticulous dissection along the lesion margin avoids intra-lesional rupture and massive intraoperative bleeding, and completely resection of multi-muscular invasive lesions can effectively reduce the postoperative recurrence rate. For the limb vascular abnormalities with intramuscular involvement, the extent of intramuscular involvement and the risk of postoperative dysfunction are core considerations in surgical decision-making. We will select the lesions with limited intramuscular involvement (such as the 19 intramuscular lesions in our study, most of which were well-delineated), as a candidate for surgical resection, to avoid damage to surrounding normal tissues, thereby reducing the risk of dysfunction. For lesions with extensive intramuscular infiltration and high risk of dysfunction, we will recommend alternative treatment strategies (such as staged interventional embolization combined with sclerotherapy) or conservative follow-up, rather than empirical radical surgical resection.

In our cohort, the total incidence of complications was 11.4%, and the recurrence rate was 5.7%. Based on postoperative pathological findings, the 35 enrolled children with limb vascular anomalies were categorized into four subgroups: venous malformations, arteriovenous malformations, lymphangiomas, and intramuscular hemangiomas. Subgroup comparative analysis revealed no statistically significant difference in postoperative recurrence rates among the four groups. However, recurrent cases occurred exclusively in the venous malformation subgroup, with a recurrence rate of 14.3% (2/14), whereas no recurrent cases were observed in the remaining three subtypes. This subtle disparity suggests that venous malformations may inherently harbor a higher recurrence propensity, presumably owing to their ill-demarcated anatomical boundaries, diffuse venous sinus distribution, and locally infiltrative growth pattern. Previous literatures report variable recurrence rate following surgical resection for upper-extremity vascular malformations. Hu et al. (21), Gasparella et al. (22) and van Doesburg et al. (23) reported a recurrence rate of 30%, 37.9% and 32% respectively. The recurrence rate of 11.4% in our cohort is comparatively lower. Notably, both recurrent cases in our study occurred in digital lesions (one hand lesion, and one toe lesion). This finding indicates that vascular malformations located in the hands and feet may be more susceptible to postoperative recurrence, highlighting the need for heightened clinical vigilance and meticulous complete resection to eliminate residual lesions.

With regard to postoperative complications, the complication, skin necrosis, neurological complications and gangrene were considered as the most severe complications. Fortunately, none of these severe complications occurred in our patients. Our complication rate (11.4%) was lower than previously reported rates documented by Hu et al. (21) and van Doesburg et al. (23) (20.9% VS 26%). Such discrepancies may be attributed to variable demographic and clinical characteristics across different studies, particularly the higher proportion of lower limb lesions enrolled in our cohort. In addition, although the overall intergroup difference in complication rates did not reach statistical significance, arteriovenous malformations and lymphangiomas exhibited numerically higher complication risks relative to venous malformations and intermuscular hemangiomas. This trend is likely attributable to their complex anatomical structure, hypervascularity, and extensive soft-tissue invasion, which inevitably increase the difficulty of surgical dissection and further elevate the risk of perioperative complications.

Notably, the limited sample size and uneven case distribution across subgroups inevitably compromised statistical power, which may account for the lack of significant between-group differences despite visible numerical disparities. Future large-sample cohort studies are therefore warranted to further validate the prognostic and safety differences among distinct pathological subtypes of pediatric limb vascular abnormalities.

This study is a single center retrospective study with a small sample size, which may lead to selection bias and limit the representativeness of the research results; Substantial variability existed in postoperative follow-up duration, and some children are followed up for a short period of time. Long-term follow-up is crucial for evaluating the long-term efficacy and safety of surgical resection. In addition, this study excluded cases of syndrome-associated vascular malformations, due to the limited clinical experience of our center in the diagnosis and management of such complex lesions.

Multi-center large-sample prospective studies are warranted to further explore clinical characteristics, diagnostic criteria and individualized treatment regimens for children with limb vascular abnormalities. Moreover, establishing a standardized long-term follow-up system to monitor prognostic outcomes and long-term complications can provide more robust clinical evidence for the standardized diagnosis and management of this disease.

## 5 Conclusion

The main clinical manifestations of vascular abnormalities in pediatric limbs include local masses and pain. Surgical resection can be adopted for localized, well-demarcated limb vascular malformations as well as cases failed to interventional therapy, yielding promising clinical outcomes with a low recurrence rate and reliable preservation of limb function.

## Data Availability

The original contributions presented in the study are included in the article/Supplementary Material, further inquiries can be directed to the corresponding author/s.
